# Evaluation of tandem repeats for MLVA typing of *Streptococcus uberis *isolated from bovine mastitis

**DOI:** 10.1186/1746-6148-2-33

**Published:** 2006-11-17

**Authors:** Florence B Gilbert, Angélina Fromageau, Jérémy Lamoureux, Bernard Poutrel

**Affiliations:** 1 INRA, UR1282, Infectiologie Animale et Santé Publique IASP, F-37380 Nouzilly, France

## Abstract

**Background:**

*Streptococcus uberis *is a common cause of bovine mastitis and recommended control measures, based on improved milking practice, teat dipping and antibiotic treatment at drying-off, are poorly efficient against this environmental pathogen. A simple and efficient typing method would be helpful in identifying *S.uberis *sources, virulent strains and cow to cow transmission. The potential of MLVA (Multiple Loci VNTR Analysis; VNTR, Variable Number of Tandem Repeats) for *S. uberis *mastitis isolates genotyping was investigated.

**Results:**

The genomic sequence of *Streptococcus uberis *(strain 0104J) was analyzed for potential variable number tandem repeats (VNTRs). Twenty-five tandem repeats were identified and amplified by PCR with DNA samples from 24 *S. uberis *strains. A set of seven TRs were found to be polymorphic and used for MLVA typing of 88 *S. uberis *isolates. A total of 82 MLVA types were obtained with 22 types among 26 strains isolated from the milk of mastitic cows belonging to our experimental herd, and 61 types for 62 epidemiologically unrelated strains, i.e. collected in different herds and areas.

**Conclusion:**

The MLVA method can be applied to *S. uberis *genotyping and constitutes an interesting complement to existing typing methods. This method, which is easy to perform, low cost and can be used in routine, could facilitate investigations of the epidemiology of *S. uberis *mastitis in dairy cows.

## Background

*Streptococcus uberis *is an important cause of mastitis in modern dairy herds. It is responsible for a significant proportion of clinical and subclinical infections in both lactating and nonlactating cows [1]. *S. uberis *is considered as an environmental mastitis pathogen in that it has a ubiquitous and widespread distribution in the cow's environment. Indeed, *Streptococcus uberis *can be isolated from bedding and pasture, as well as intramammary and extramammary sites on the cow [2,3]. This may contribute to the poor efficiency of recommended mastitis control measures toward this pathogen, such as teat dipping and intramammary antimicrobial therapy at the end of each lactation period. The main route of transmission appears to be from environmental sources but recent epidemiological studies suggest that cow-to-cow transmission is likely to be occurring [4,5].

Understanding the different sources, the dynamics of spread and transmission of *S. uberis *is a prerequisite for the development of mastitis control programs. In an attempt to differentiate *S. uberis *strains, a number of typing methods have been developed, among them restriction endonuclease fingerprinting, random amplified polymorphic DNA (RAPD), repetitive extragenic palindromic (REP)-PCRs and DNA macrorestriction analysis by pulse-field gel electrophoresis (PFGE) [4,6,7]. All these methods enlighted the high genomic diversity of *S. uberis *strains but most of them display limitations in terms of discriminatory power, standardization and reproducibility. PFGE is reliable, reproducible and highly discriminatory but its use in large epidemiological studies and routine surveillance is limited because it is technically demanding, time-consuming, expensive and complex DNA patterns may be difficult to interpret, especially for large collections of isolates [8]. Recently, two multilocus sequence typing (MLST) systems were described for *S. uberis *subtyping [9,10]. The first one, based on four housekeeping genes and two virulence genes, was evaluated with 50 *S. uberis *isolates and compared to ribotyping and RAPD typing. MLST was superior to the other techniques in terms of discriminatory power, concordance with epidemiological data, and quantitative information regarding relatedness of isolates [9]. The other MLST system relies on the use of sequences from seven housekeeping gene fragments. It was used to study *S. uberis *isolates from United Kingdom and New Zealand. These two populations were shown to be distinct and three major clonal complexes with different geographic prevalences have been identified. This method is suitable for the analysis of *S. uberis *population structure and evolutionary relationships and a database has been constructed on a central MLST Web site [10,11]. However, MLST may not be suitable for routine surveillance and local epidemiological studies because of the high cost and the necessity of access to a high-throughput DNA sequencing facility.

Polymorphic tandem repeat typing is a new generic technology which has been proved to be very efficient for bacterial pathogens such as *B. anthracis*, *M. tuberculosis*, *P. aeruginosa*, *Y. pestis *and is compatible with the development of internet-based resources [12]. It takes advantage of the release of genome sequence data for the identification of tandem repeats (TRs). The development of an assay then requires the evaluation of tandem repeats polymorphism, i.e. tandem repeats showing interindividual length polymorphism, on well-selected sets of isolates. By using different polymorphic tandem repeats (Variable Number Tandem Repeats), it is possible to discriminate strains for a low cost with ordinary molecular biology equipment and the data can be easily exchanged and compared [12]. This method, called Multiple Locus VNTR Analysis (MLVA), has been recently applied to several bacterial species including *Staphylococcus aureus *[13,14] and *Escherichia coli O157:H7 *[15]. MLVA can evolve with resources as fragment size, repetition number of tandem repeat can be determined by capillary electrophoresis with a DNA analyzer, as well as nucleotidic sequence.

The aim of the present study was to identify polymorphic tandem repeats in *S. uberis *genomes that could be included in a MLVA procedure able to differentiate isolates and to contribute to the comprehension of *S. uberis *epidemiology and ecology in dairy herds.

## Results and discussion

The genome sequence of *S.uberis *strain 0140J (isolated from a case of bovine mastitis) was explored with the Tandem Repeats Finder software using the advanced version with default parameters. In these conditions, twenty-seven tandem repeats (TRs) with a maximal unit length of 208 bp were detected around the chromosome. Two tandem repeats displaying a period size smaller than 12 bp were not considered in this study, although one of them is comprised in TR16. This choice was motivated by the relative ease with which allelic differences can be resolved by agarose gel electrophoresis. Among the 25 TRs studied, the lowest value of nucleotide sequence identity between individual repeat units was 72% and copy numbers of repeat units in *S. uberis *0140J genome varied from 2.0 to 5.5 (Table 1). Twelve TRs had a repeat unit length with multiples of 3 and all of them, except TR13, were embedded in predicted open reading frames (ORFs) according to the current preliminary gene prediction and *S. uberis *BLAST server. TRs 8 and 13 were partially contained in ORFs and displayed the start and stop codons, respectively, while TR12 contained a putative small ORF. Other TRs were localized in non-coding regions of the genome.

A set of 24 epidemiologically unrelated *S. uberis *isolates, i.e. collected at different times in herds from diverse French areas, were used to evaluate the size polymorphism of the TRs by PCR with specific primers. Overlapping or adjoining TRs, namely TRs 1 and 2, 6 and 7, 11 and 12, and 16 and 17 were co-amplified with a single set of primers (Table 2). A total of 9 TRs were found to be polymorphic as diverse size amplicons were obtained but two of them (TR16+17 and TR25) were amplified for a minority of isolates. These TRs were not investigated further. The seven polymorphic TRs for which amplicons were obtained for all isolates were used to type the entire set of strains. These polymorphic TRs are the followings: TRs 10, 11+12, 15, 18, 20, 21 and 24. With the exception of TR20 which may be part of an ORF (SUB1226, undefined product), all polymorphic TRs were localized in intergenic regions. Among the monomorphic TRs, TR6+7 and TR 9 were not amplified in all isolates. The inability to amplify TR6+7, TR9, TR16+17 or TR25 for some strains may be due to a local genetic variability in the area annealing with primers or to the absence of the corresponding sequences in their genomes.

**Figure 1:**
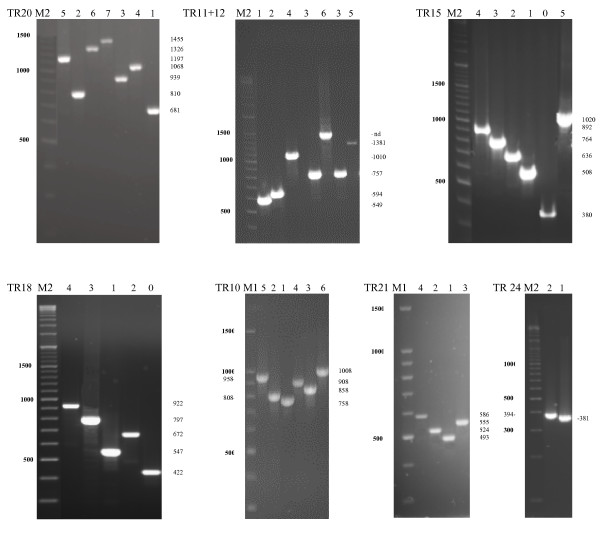
**Representative agarose gel electrophoresis of PCR-amplified VNTR**. Lane M1: 100 bp DNA ladder; lane M2: 100 bp+500 bp DNA ladder. Numbers on top of other lanes designate alleles expressed as number of repeats, except for TR11+12. According representative amplicons sequences of TR11+12, alleles 1 to 5 corresponded to 2 TR12, 1 TR11 and 1 TR12, 1 TR11 and 2 TR12, 3 TR11 and 1 TR12, 4 TR11 and 2 TR12, respectively. The predicted size in bp of alleles, calculated by sequencing of representative amplicons and *S. uberis *0140J genome sequence data, is indicated. Nd: the predicted size of this allele was not calculated because the sequence of the corresponding amplicon was incomplete.

MLVA typing of the entire set of 88 *S. uberis *isolates was performed with the seven selected TRs. The set of alleles obtained with these TRs is presented in Figure 1. A total of 82 MLVA types were obtained with 22 types among the 26 strains isolated from the milk of cows belonging to our experimental herd, and 62 types for the 63 epidemiologically unrelated strains, i.e. collected in different herds and areas (Table 3). With the exception of the *S. parauberis *strain NCDO 2020, all the isolates used in the study were defined as *S. uberis *according to PCR amplification results using primers targeting species-specific parts of the 16S rRNA gene as previously described [16]. This confirmed that the occurence of *S. parauberis *as causative agent of bovine mastitis appears to be rare [16,17]. The *S. parauberis *strain NCDO 2020 was the sole strain for which no amplification occurred with most of the TRs (Table 3). This result is probably the consequence of some genetic variability and it would be interesting to determine if this variability is specific to the NCDO 2020 strain or if it represents a general feature of *S. parauberis *genotype. Concerning other major *Streptococcus *species implicated in bovine mastitis, i.e.*Streptococcus agalactiae *and *Streptococcus dysgalactiae*, none of the 7 polymorphic TRs were amplified from the genomic DNA preparations of the 6 strains that we tested (data not shown). Moreover, no significant alignments of the 7 TRs sequences with available *S. agalactiae *genomic sequences were found when a Blast analysis was performed.

The MLVA system described in this study displayed a satisfactory discriminatory power as it was able to distinguish most of the *S. uberis *isolates collected in different herds or from the cows belonging to our experimental herd (Table 3). These results are in agreement with previous studies that showed, using diverse typing methods, the high genetic variability of *S. uberis *strains [4,6,7,18]. The 26 isolates collected from 17 of the cows in our experimental herd were divided into 22 MLVA types with 2 types (8 and 72) represented by 3 isolates collected from the milk of different cows. Strains isolated simultaneously from different quarters of individual cows harboured different MLVA types. These observations are congruent with previous reports stating that the environment rather than infected mammary glands was the likely source of *S. uberis *infections, even if cow to cow transmission could occur [4,5,18]. Overall, the 88 *S. uberis *strains used in this study were classified in 82 MLVA types among them 2 (types 3 and 49) comprised 2 strains that were isolated in different herds and years. Even if some *S. uberis *strains isolated in different herds and countries have already been demonstrated to belong to the same type using methods such as PFGE [4] or MLST [9,10], the strains belonging to MLVA types 3 and 49 may be distinguished with another typing method or additional TRs. Indeed, *S. uberis *strains harbouring the same alleles in a MLST study appeared to be different using PvuII ribotyping and some strains belonging to the same PvuII ribotype were considered different using MLST [9]. The MLVA system described here has already a high discriminatory power but, considering the variability of *S. uberis *genotype, it would be interesting to look for additional polymorphic TRs to complete this set. Among the 7 TRs used, TR11+12 and TR15 were the most efficient with a Simpsons diversity index of 0.787 and 0.748, respectively. Sequencing of amplicons corresponding to TR11+12 showed that TR11 and TR12 are both polymorphic and that some strains may harbour only one of them. Indeed, the sequence analysis of one representative of allele 1 revealed that TR11 was not contained in the genome of the corresponding strain. TR 21, TR20 and TR18 exhibited a Simpsons diversity index value of 0.677, 0.671 and 0.629, respectively. TR10 (diversity index = 0.590) and TR24 (diversity index = 0.363) were the less discriminatory. Indeed, TR24 displayed only 2 alleles with our strains and TR10 allowed us to define 6 alleles but most of the strains possessed the allelic forms 3 or 4.

## Conclusion

This investigation validates the usefulness of the MLVA typing method for bovine *S. uberis *strains and a first set of markers. The proposed MLVA system can be evaluated with collections of strains from diverse geographical origins, completed by additional polymorphic tandem repeats and compared with other typing methods.

In contrast to PFGE or MLST, the MLVA method is easy and fast to perform, low cost and could constitute the method of choice for short-term epidemiological studies when population structure and evolutionary relationships are not a concern. Investigations at the herd level such as analysis of outbreaks, strains implicated in persistent infections or demonstrating different antibiotic treatment susceptibilities, should be facilitated and allow the development of adapted herd management measures.

## Methods

### Strains and DNA isolation

A total of 87 *S. uberis *isolates were selected from our collection for this study. Sixty-one strains were isolated between 1961 and 2004 from the milk of 61 mastitic dairy cows in 59 dairy herds localized in 23 departments of France and 26 strains were isolated in 2003 and 2004 from the milk of 17 mastitic cows belonging to our experimental dairy herd. In addition, *S. uberis *ATCC 9927 and *S. parauberis *NCDO 2020 were included in our study. All strains were identified as *S. uberis *using conventional microbiological techniques as previously described [16,18]. The identification of the species *S. uberis *and *S. parauberis*, that are phenotypically indistinguishable, was performed by PCR using primers targeting species-specific parts of the 16S rRNA gene as previously described [16].

DNA was isolated with the Wizard Genomic DNA Purification Kit (Promega) according to the instructions of the manufacturer, except that the lysis of the bacteria was performed with lysozyme and mutanolysin (Sigma).

### Identification of tandem repeats

The genomic sequence of *S. uberis *0140J was produced by the *S. uberis *Genomic Group at the Sanger Institute [19]. This sequence was analyzed by using the Tandem Repeats Finder software with default parameters [20,21]. Tandem repeats displaying a period size smaller than 12 bp were not considered. The program generates an output file showing the repeat location, the repeat length and copy number, the nucleotidic composition of the repeat and the sequence of flanking regions. Tandem repeats were named according to their chromosomal locations (Table 1). As annotation of *S. uberis *sequence is ongoing, the putative intragenic or extragenic position of the TRs was determined with the *S. uberis *BLAST server [19].

### VNTR amplification and genotyping

Primer sets (Table 2) were designed to anneal within flanking regions of the VNTRs using the software Primer3 [22]. Adjoining and overlapping VNTRs were amplified with a unique primer set. The PCR was performed with 10 ng of *S. uberis *genomic DNA in a 25-μl reaction mixture containing 1X PCR buffer, 0.75 U of UptiTherm DNA polymerase (Interchim), 200 μM dNTPs (Promega), 1.5 mM MgCl_2_, and 0.5 μM of the forward and reverse primers. The amplifications were carried out in a PTC-100 MJ Research thermocycler with the following program: 1 step of 5 min at 94°C followed by 30 cycles of 30 s at 94°C, 45 s at 52°C except for VNTRs 11–12 for which the hybridation step was carried out at 44°C, 1 min at 72°C, and finally 1 step of 5 min at 72°C.

Next, 8 μl of each amplicon was electrophoresed in a 1.5% agarose gel (Amresco) in the presence of ethidium bromide in 0.5X TBE buffer (Tris-borate-EDTA) (Amresco). O'Range Ruler™ 100 bp+500 bp (MBI Fermentas, Vilnius, Lithuania) and Bench Top 100 bp DNA Ladder (Promega) were used as size standards. The DNA bands were visualized on a UV transilluminator and analyzed with an Alpha Imager Gel Analysis System Fluorchem (Alpha Innotech Corporation) and by eye. Precise size of amplicons and number of repeats were deduced after sequencing of representative PCR products by using forward and reverse primers (Genome Express, Meylan, France) and by comparison of the obtained sequences to *S. uberis *0104J genome (Table 1). For TR11+12, which consists of 2 adjoining TRs amplified with a single set of primers, allele numbers were attributed independently of the number of repeats, as only one representative of different size PCR products was sequenced. To facilitate multiple gel analysis and allele number attribution, previously defined alleles were included in each experiment. The Simpsons diversity index of each TR was determined via the online tool V-DICE available at the Health Protection Agency website [23]. Values of this index can range from 0 (no diversity) to 1 (complete diversity).

## Abbreviations

VNTR; Variable Number of Tandem Repeats

MLVA; Multiple locus VNTR Analysis

TR; Tandem Repeat

## Competing interests

The author(s) declare that they have no competing interests.

## Authors' contributions

AF and JL prepared the DNA samples and did most of the typing work. FBG initiated and managed the project, analyzed *S. uberis *genome for tandem repeat searches, designed primers and wrote the manuscript. All authors read and approved the final manuscript.

## Figures and Tables

**Table 1:** List of tandem repeats investigated

Tandem repeat (TR)	Repeat length (bp)	Copy number in 0104J genome	Percent* matches	Location in 0140J genome
TR 01	18	2.2	86	26862–26900
TR 02	12	5.5	72	26850–26909
TR 03	124	3.2	98	38018–38419
TR 04	32	2.0	93	73534–73597
TR 05	18	2.2	86	79094–79132
TR 06	129	2.2	89	167675–167961
TR 07	129	2.4	86	167708–168016
TR 08	29	2.2	100	242079–242143
TR 09	29	2.4	80	281367–281436
TR 10	50	4.0	95	423156–423356
TR 11	208	2.0	96	591209–591624
TR 12	163	2.0	93	591626–591951
TR 13	21	2.0	100	654394–654434
TR 14	28	2.1	93	902276–902335
TR 15	128	2.0	94	992075–992330
TR 16	18	3.3	90	1066925–1066983
TR 17	27	2.9	80	1067084–1067161
TR 18	125	3.0	97	1136470–1136844
TR 19	16	2.0	93	1147017–1147048
TR 20	129	3.0	97	1209337–1209723
TR 21	31	2.0	97	1240441–1240502
TR 22	12	2.5	94	1345878–1345907
TR 23	12	2.5	88	1494746–1494775
TR 24	13	2.0	100	1496960–1496985
TR 25	15	3.9	93	1813855–1813913

* percent of matches between adjacent copies.

**Table 2:** Primers used in this study

VNTR	Primer sequence 5'-3'	Location in 0140J strain genome
01–02	TGCAGCACAAATGGAAACTGC	26778–26798
	AGCTACAGGTGCAGGTGTTGC	27001–27021
03	CAGAATTAAAATATCCTTGTTTTTAC	37728–37753
	ATTCTTCTTCATAGTCATATAGTGGT	37942–37967
04	TGAAACTGCAACACGTACAGG	73293–73313
	ACGTTTGGAGTTGCTTCGATG	73709–73729
05	CGCAGAAAATCCTCAAGGAGC	78991–79011
	ACAGCATCACCAACACCCATG	79290–79310
06–07	CATATTACCTCTTATTCCTCTT	167562–167583
	TCAATAATATCAACAATCTCA	168054–168074
08	TTTGGATGCTTTCTTGGTTGG	242005–242025
	TTGCAGAGATTCGCCAACCTG	242325–242345
09	CTTTTAGCATAATTTTACCTTTTA	281297–281320
	ATAAGATCAATATGAAGTGTATCTA	281591–281615
10	CGAACATGGTTATATCGTCCCAG	423092–423114
	TCGGCAACAATGGCCATATCG	423980–424000
11–12	GTTTATTATCTTTATGAGTATGCTT	591027–591051
	TATCTATAAAATGCAAACGATTTA	591969–591992
13	ATGAACCGACAGCTAGTCTGG	654229–654249
	CATATGGTCGTACGGTCCCTG	654572–654592
14	CAAGCCTTGAAATTCATCTCC	902092–902112
	GCAAGGTTTTTCCTCGTTGA	902638–902657
15	AATATAGGAATTAATTTCAGTTCTTGAC	991780–991807
	TAAGTTTACCCTAGAAGAGAATACAGTT	992388–992415
16–17	ACCATTAGAGATGGTTACTTCTTTAC	1066796–1066821
	GTCTGAACGGACTTAATGGTATT	1067309–1067331
18	GAAACAAAGTTTCCATCTTTAT	1136358–1136379
	AAGATGAAGTTTATTATGGATTCTC	1137131–1137155
19	CCTCCACCTGATGGACCAACG	1146876–1146896
	CAGGTTGTCGAAGCAACTGTC	1147205–1147225
20	AAAAGAACTTTATGAATGGAAAAC	1209150–1209173
	ATGGTATCTAATAAGGTAAGACCAC	1210065–1210089
21	CAGTTGTACTTGTGGCTTGAC	1240077–1240097
	TTGCCCTTCGTTGGTTCGCTG	1240581–1240601
22	CCCCTAAAATCGCAGCAATA	1345744–1345763
	TTTGACCAATTATGGTCTATACGC	1345908–1345931
23	TTTACCTTCAGTTTCTTTAATC	1494658–1494679
	TAAAGATTTAGGTACTCAAAAAG	1494840–1494862
24	ACTTCCGAATTGTCATCGTTCG	1496796–1496817
	TGCTGACAGTAATGAACCAGC	1497170–1497190
25	GGTGTTTATTTCATATTGGTCAGC	1813638–1813661
	GAAGTTCAAAGTTGGTTCAAATGTT	1813932–1813956

**Table 3:** Characteristics of bovine *S. uberis *strains and MLVA typing results

Isolate number	Year of isolation	Geographical area	Tandem Repeats							MLVA type
				
			10	11+12	15	18	20	21	24	
1	2003	50	1	2	1	2	4	3	1	1
2	2003	37	1	5	1	1	4	3	1	2
3	2002	73	2	2	0	1	4	1	2	3
4	2004	73	2	2	0	1	4	1	2	3
5	2003	57	2	3	2	1	3	3	2	4
6	2003	15	2	4	4	2	4	1	2	5
7	2003	37	2	6	3	2	6	3	2	6
8	2003	37	3	1	3	2	5	3	2	7
9	2003	37	3	1	4	2	5	3	2	8
10	2003	37	3	1	4	2	5	3	2	8
11	2003	37	3	1	4	2	5	3	2	8
12	1993	37	3	2	4	2	2	1	2	9
13	2001	61	3	3	1	0	5	1	2	10
14	2003	72	3	3	1	1	3	1	1	11
15	2003	56	3	3	1	1	4	2	2	12
16	1997	08	3	3	2	0	6	1	1	13
17	2003	53	3	3	2	2	3	1	1	14
18	2004	74	3	3	2	2	3	1	2	15
19	2004	74	3	3	2	2	4	4	1	16
20	2003	37	3	3	3	1	4	1	1	17
21	2003	37	3	3	3	1	4	4	1	18
22	2003	37	3	3	3	1	5	1	1	19
23	2003	44	3	3	3	2	4	1	2	20
24	2000	02	3	4	2	0	3	1	2	21
25	2003	35	3	4	2	2	3	1	1	22
26	2003	37	3	4	3	3	4	3	2	23
27	2003	63	3	5	2	1	5	2	2	24
28	2003	37	3	5	2	2	4	3	2	25
29	2003	37	3	5	2	3	4	3	2	26
30	2003	15	3	5	3	1	5	3	1	27
31	2003	62	3	5	4	2	5	1	2	28
32	1981	37	3	6	1	3	4	2	1	29
33	1982	37	3	6	1	3	4	2	2	30
34	2003	62	4	1	0	1	4	2	2	31
35	2003	37	4	1	3	3	7	2	2	32
36	2003	37	4	1	4	2	5	3	2	33
37	2002	80	4	2	1	2	1	1	1	34
38	2003	37	4	2	3	1	5	4	2	35
39	2003	44	4	2	5	1	4	1	1	36
40	2003	62	4	3	1	2	4	1	2	37
41	2003	53	4	3	1	3	4	2	2	38
42	2003	08	4	3	2	2	7	3	2	39
43	1988	37	4	3	2	3	4	1	2	40
44	2003	64	4	3	3	2	3	2	2	41
45	2003	35	4	3	3	2	4	2	2	42
46	2003	37	4	3	3	2	7	3	2	43
47	2003	62	4	3	3	3	4	1	2	44
48	2003	80	4	3	4	2	4	2	2	45
49	2003	37	4	3	4	2	4	3	2	46
50	2003	64	4	4	1	3	7	2	1	47
51	2003	62	4	4	1	4	5	1	2	48
52	2003	15	4	4	2	2	4	2	2	49
53	2003	37	4	4	2	2	4	2	2	49
54	2003	56	4	4	3	1	4	1	2	50
55	2003	37	4	4	3	2	4	2	2	51
56	2003	62	4	4	3	3	4	2	2	52
57	2003	37	4	4	4	2	4	3	2	53
58	2003	85	4	4	4	2	7	2	2	54
59	2003	62	4	5	1	1	4	2	2	55
60	2004	73	4	5	1	2	3	2	2	56
61	2003	62	4	5	1	3	4	1	2	57
62	2003	80	4	5	1	3	5	1	2	58
63	2003	62	4	5	2	1	2	2	2	59
64	1961	89	4	5	2	2	4	2	2	60
65	2004	74	4	5	2	2	4	3	2	61
66	2003	08	4	5	2	2	7	2	2	62
67	2003	62	4	5	2	3	3	4	2	63
68	2003	80	4	5	2	3	4	2	2	64
69	1995	76	4	5	3	2	3	2	2	65
70	2003	64	4	5	3	2	4	2	2	66
71	2003	37	4	5	3	2	5	2	2	67
72	2003	37	4	5	3	2	6	2	2	68
73	2003	80	4	5	3	3	4	2	1	69
74	2003	80	4	5	3	3	4	2	2	70
75	2003	80	4	5	3	4	2	2	2	71
76	2003	37	4	6	2	2	4	2	2	72
77	2002	37	4	6	2	2	4	2	2	72
78	2003	37	4	6	2	2	4	2	2	72
79	2003	37	4	6	2	2	5	2	2	73
80	2003	35	4	6	3	2	4	2	2	74
81	2003	72	4	6	4	2	4	3	1	75
82	2003	08	4	6	4	3	5	2	1	76
83	2003	08	5	3	2	1	5	1	2	77
84	1992	35	5	3	2	2	4	1	1	78
85	2003	08	6	3	2	2	4	1	2	79
86	2003	15	6	3	3	1	3	1	1	80
87	2003	15	6	4	2	2	3	1	1	81
ATCC 9927	1971	Etats-Unis	3	3	2	2	5	1	2	82
NCDO 2020			3	-	-	-	-	-	-	

-, no amplification.

Alleles represent numbers of repetitions deduced from sequence analysis of representative amplicons, except for TR11+12.

All the strains isolated in 2003 and 2004 in the geographical area 37 were collected from the milk of cows belonging to our experimental herd.

ATCC 9927 and NCDO 2020 are *S. uberis *and *S. parauberis *reference strains, respectively.
